# The Effect of Accelerated Aging on the Molecular Weight and Thermal and Mechanical Properties of Polyester Yarns Containing Ceramic Particles

**DOI:** 10.3390/polym15061348

**Published:** 2023-03-08

**Authors:** Gabriela Mijas, Marta Riba-Moliner, Diana Cayuela

**Affiliations:** 1 Terrassa Institute of Textile Research and Industrial Cooperation (INTEXTER), Universitat Politècnica de Catalunya (UPC), 08222 Terrassa, Spain; gabriela.mijas@upc.edu (G.M.);; 2 Fundación Asociación de Becarios Retornados EC (ABREC), Quito 170518, Ecuador; 3 Department of Materials Science and Engineering (CEM), Universitat Politècnica de Catalunya (UPC-ESEIAAT), 08222 Terrassa, Spain

**Keywords:** accelerated aging, polyethylene terephthalate (PET), multifilament yarns titanium dioxide (TiO_2_), silicon carbide (SiC), fluorite (CaF_2_)

## Abstract

The accelerated aging of polyethylene terephthalate (PET) multifilament yarns containing nano or microparticles of titanium dioxide (TiO_2_), silicon carbide (SiC), or fluorite (CaF_2_) at a maximum percentage of 2% has been studied. For this, the yarn samples were introduced into a climatic chamber at 50 °C, 50% relative humidity, and an ultraviolet A (UVA) irradiance of 1.4 W/m^2^. They were then removed from the chamber after periods of between 21 and 170 days of exposure. Subsequently, the variation in weight average molecular weight, number molecular weight, and polydispersity was evaluated by gel permeation chromatography (GPC), the surface appearance was evaluated using scanning electron microscopy (SEM), the thermal properties were evaluated using differential scanning calorimetry (DSC), and the mechanical properties were evaluated using dynamometry. The results showed that, at the test conditions, there was degradation in all of the exposed substrates, possibly due to the excision of the chains that make up the polymeric matrix, which resulted in the variation in the mechanical and thermal properties depending on the type and size of the particle used. This study provides insight into the evolution of the properties of PET-based nano- and microcomposites and might be helpful when selecting materials for specific applications, which is of great interest from an industrial point of view.

## 1. Introduction

The incorporation of a small amount of ceramics into a polymer yields a composite material with properties such as hardness, stiffness, heat resistance, UV resistance, water absorption, gas permeability, etc., at a low cost. For this reason, the development of functional fibers will be a key factor for the textile industry and, of particular interest, those containing ceramic powders. The use of nanotechnology can enhance the use of multifunctional textiles and provide them with new features, such as antibacterial, fungicidal, or water-repellent properties [1,2,3].

Some ceramic particles have been used from a textile point of view due to the multiple advantages they could bring to a substrate thanks to their intrinsic properties. Titanium dioxide (TiO_2_), because of its photocatalytic properties, provides self-cleaning properties and UV protection as well as an antibacterial barrier [4,5,6,7,8]. Calcium fluoride (CaF_2_) is of interest for its high stability, nonhygroscopic behavior, and transparency [9,10] and silicon carbide (SiC) has low density, high melting and decomposition temperatures, good mechanical and thermal properties, and excellent chemical and oxidation stabilities that have led to its study as a UV-blocking agent [11,12,13].

In this context, in previous studies carried out by the research team, the effect of the inclusion of nano and microparticles of TiO_2_, SiC, and CaF_2_ on the physical and chemical properties of polyethylene terephthalate (PET) has been analyzed. These results have made it possible to determine the most suitable processing conditions and dispersant for obtaining the PET multifilament yarns used in this work [14,15,16,17,18,19,20].

Polyethylene terephthalate, as well as other polymeric substrates, is susceptible to being affected by climatic conditions, which have destructive effects on the durability and performance of these materials. Among the factors that can be mentioned are solar radiation, humidity, and temperature [21,22]. The degradation process is executed in two simultaneous ways: yellowish discoloration and the loss of mechanical properties. Damage occurs when photons of light interact with the molecular chains that constitute the polymer structures. Shorter wavelengths possessing higher photon energies are more strongly absorbed in most polymeric materials and are more likely to break their chemical bonds. Because ultraviolet radiation has more photons of energy, it can break more types of bonds [23,24,25].

The objective of this research was to study the influence of the presence of nano or microparticles of TiO_2_, SiC, or CaF_2_ in the accelerated aging (50 °C, 50% RH, 1.4 W/m^2^ UVA) of PET multifilament yarns. For this, molecular weight and polydispersity variation were evaluated by gel permeation chromatography (GPC), the surface appearance by scanning electron microscopy (SEM), the thermal properties by differential scanning calorimetry (DSC), and the mechanical properties by dynamometry.

## 2. Materials and Methods

### 2.1. Materials

The study was carried out on POY multifilament yarns fabricated and supplied by IQAP Masterbatch Group S.L. (Masies de Roda, Spain). One hundred percent polyester (extra-bright) textile quality pellets were provided by ANTEX, S.L. (Anglès, Spain). The type and quantity of particles present in each yarn as well as the amount of dispersant Licowax-E (montane acid ester with multifunctional alcohols) supplied by Clariant (Sant Joan Despí, Spain) used, are presented in Table 1. 

**Figure 1:**
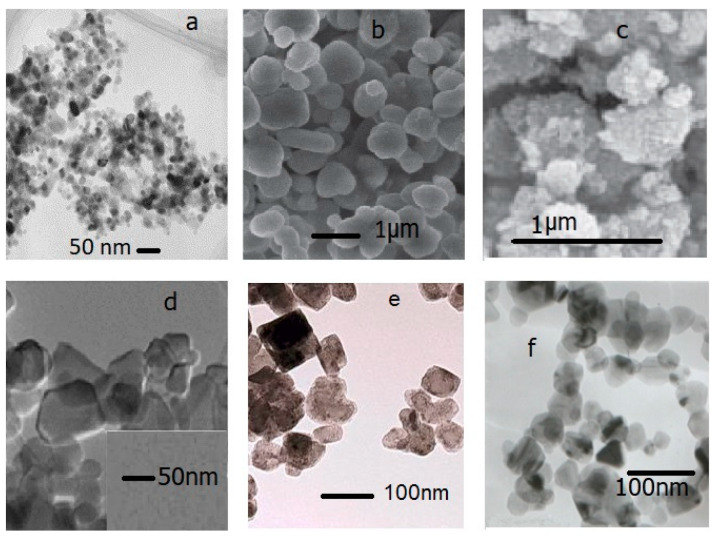
TEM micrographs of raw materials: (**a**) TiO_2_ rutile nm [26], (**b**) TiO_2_ rutile µm [27], (**c**) TiO_2_ anatase nm [28], (**d**) TiO_2_ anatase µm [29], (**e**) CaF_2_ nm, and (**f**) SiC nm [30].

TiO_2_ particles were supplied by Zeus Química S.A. in rutile and anatase crystalline forms of nano and micrometer sizes, and their characteristics are shown in Table 2. CaF_2_ nanoparticles were synthesized with the methodology described in previous work [20] and their mean particle size obtained by dynamic light scattering (DLS) was 110 nm. β-SiC spherical nanoparticles, with a purity of 97.5%, an average particle size of 45–55 nm, and a specific surface area (BET) of 35–40 m^2^/g were purchased from NanoAmor (Houston, United States). TEM micrographs of raw materials are presented in Figure 1.

### 2.2. Post-Spinning Stretching

All substrates were subjected to a 1:2 stretching to provide them with optimum textile properties. The material infeed and outfeed speeds were 10 m/min and 20 m/min, respectively, on the corresponding drafting rolls.

### 2.3. Accelerated Aging

Thirty multifilament yarns of each substrate were placed parallel to each other on a sample holder grid. The substrates thus arranged were treated in a climatic chamber Binder GmbH 240 KBF ICH (E5) at conditions inside the equipment of 50 °C, 50% relative humidity, and 1.4 W/m^2^ ultraviolet A (UVA) exposure. To establish the treatment conditions, the “humidity–temperature with light” diagram available in the equipment specification sheet was used. Both temperature and humidity were within the standard range. The yarns were taken out of the chamber after 21, 35, 77, 91, 105, 119, 133, 148, and 170 days of exposure.

### 2.4. Washing

Once removed from the climatic chamber, the substrates were washed with a 1 g/L Hostapal UH liquid surfactant solution (Archroma, Sant Joan Despí, Spain) at 35 ± 2 °C for 30 min with a 1:30 liquor ratio to remove surface impurities. Afterward, the substrates were rinsed with distilled water at 35 ± 2 °C for 5 min and then rinsed a further three times with distilled water at room temperature for 5 min each time. Once the yarns were washed, they were left to dry at room temperature.

### 2.5. Molecular Weight 

The procedure described in Gacén [31] was followed. Approximately, 3 mg of washed yarns were placed in a test tube with 0.2 mL of o-chlorophenol >99% (Sigma-Aldrich, Steinheim, Germany). The tubes were placed in a polyethylene glycol bath at 80 °C for 30 min. Once the yarn was dissolved and the tubes cooled, 1.8 mL of chloroform (HPLC grade, stabilized with amylene, approx. 150 ppm, Scharlau, Barcelona, Spain) was added. The mixture was stirred and then filtered (PTFE filter, 0.45 µm) and transferred to a vial. Analysis was carried out in a high-performance liquid chromatograph (Perkin Elmer, Norwalk, CN, USA) provided with an ISS200 injector, 200lc series pump, an oven for series 200 columns, and a series 200a refractive index detector. The test was performed at 40 °C with HPLC grade chloroform (Scharlau, Barcelona, Spain) at a flow rate of 1 mL/min on a PLgel 5 µm 300 × 7.5 mm Mixed-D column (Agilent, Stockport, UK) and proceeded using an Oligopore Column 6 µm 300 × 7.5 mm (Agilent, Stockport, UK). The system was calibrated with polystyrene standards with weight average molecular weight in the range of 2350–200,000 g/mol (Waters, Milford, CN, USA). TotalChrom Navigator—TurboSEC v. 6.3.1.0504 software (Perkin Elmer, Norwalk, CN, USA) was used to calculate molecular weight in number, molecular weight in weight, and polydispersity.

### 2.6. Fourier Transform Infrared Analysis-Attenuated Total Reflection (FTIR–ATR)

A Nicolet 6700 FT–IR spectrometer (Thermo Scientific, Waltham, MA, USA) with a SmartOrbit Diamond ATR accessory was used to determine the functional group changes during aging. Thirty-two scans were completed in the 400–4000 cm^−1^ range, at a spectral resolution of 2 cm^−1^.

### 2.7. Thermal Analysis (Differential Scanning Calorimetry)

Samples of 4–5 mg sealed in an aluminum crucible were analyzed in a DSC 7 differential scanning calorimeter (Perkin Elmer, Norwalk, CN, USA). The studies were carried out under the following conditions: (i) holding at 40 °C for 5 min, (ii) heating from 40 °C to 300 °C at 20 °C/min, (iii) holding at 300 °C for 5 min, (iv) cooling from 300 °C to 40°C at 20 °C/min, (v) holding at 40 °C for 5 min, and (vi) heating from 40 °C to 300 °C at 20 °C/min. Nitrogen (N_2_) was used as purge gas at a flow rate of 35 mL/min. Samples were analyzed with Pyris 5.00.02 software (Perkin Elmer, Norwalk, CN, USA) and the results are the average of two determinations.

### 2.8. Lineal Density (Count)

Prior to testing, the yarns were conditioned at 20 ± 2 °C and 65 ± 4% RH for 24 h according to UNE-EN 13392:2001 [32]. For count determination, 100 m of each yarn was collected in a wrap reel Aspe (JBA, Cabrils, Spain) and then weighed on an analytical balance (±0.1 mg). Subsequently, the mass-to-length ratio in tex was calculated. This procedure was carried out in triplicate.

### 2.9. Tenacity and Elongation

Tensile tests were performed based on the UNE-EN ISO 2062:2010 standard [33]. A Uster Tensokid automatic PE 4056 dynamometer was used with a grip separation of 500 mm, a test speed of 500 mm/min, and an elongation of 85% of the initial specimen. TestXpert Standard v 6.01 software (ZwickRoell S.L., Barcelona, Spain) was employed to analyze the tensile properties of the yarn.

### 2.10. Micrographs

Surface changes to the yarns during the exposure in the climatic chamber were analyzed using a FEI Phenom FP3950/00 scanning electron microscope (Phenom-World, Eindhoven, The Netherlands). Previously, the samples were coated with Au/Pd in an inert Ar atmosphere for 135 s and a current of 18 mA. The distance between the samples and the coating source was 35 mm. 

## 3. Result and Discussion

**Figure 2:**
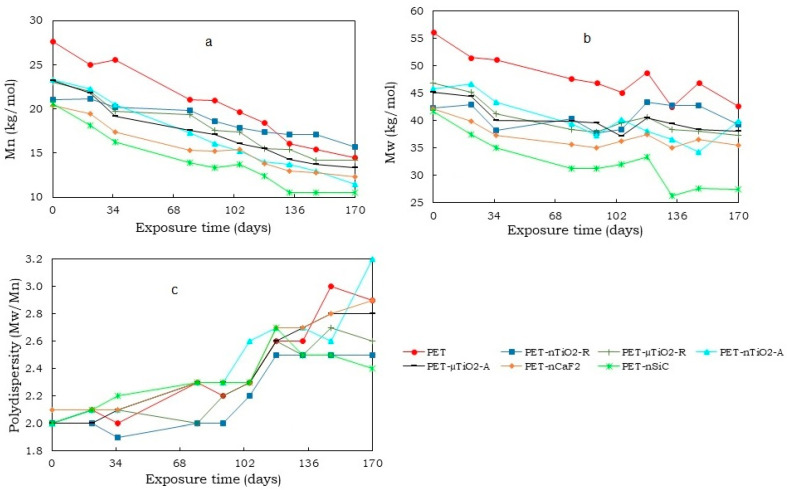
Variation in (**a**) number average molecular weight, (**b**) weight average molecular weight, and (**c**) polydispersity of yarns at different exposure times.

The variations in the average molecular weight, weight average molecular weight, and polydispersity of the yarns at different exposure times are shown in Figure 2.

**Figure 3:**
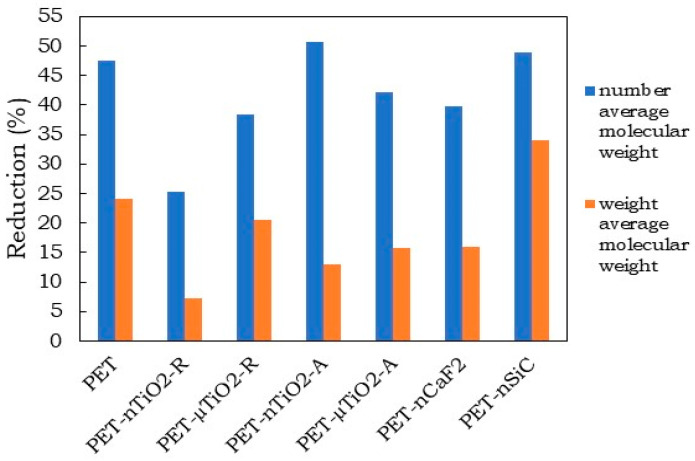
Molecular weight reduction after 170 days of exposure.

In all cases, there was a decrease in the number average molecular weight ($\bar{M_{n}}$) (Figure 2a), which could be the result of the scission of the molecular chains that constitute the polymeric matrix leading to the formation of oligomers [34]. Nevertheless, depending on the type of particle, the effect was different on the substrate when compared to pure PET. The reduction in the yarns with anatase TiO_2_ particles was greater than that with the rutile type, which could be because the rutile crystalline form has a higher ultraviolet scattering effect in comparison to the anatase form [35,36]. Similarly, a decrease in the weight average molecular weight ($\bar{M_{w}}$) (Figure 2b) values were observed, to a greater degree, in PET-nSiC. The final reduction percentages after 170 days of exposure are shown in Figure 3. In the case of PET-nSiC, degradation was observed to have stopped after 133 days of exposure in the chamber. The greatest decrease in molecular weight in number was found in PET-nTiO_2_-A, very similar to PET-nSiC, and both higher than that found for pure PET. The higher specific surface area of the nanoparticles could have caused the ultraviolet radiation to produce a larger degradation in the polymeric matrix [4,37].

Regarding the polydispersity (Figure 2c), an increase was observed over time that could be due to the scission of the molecular chains resulting from the degradation undergone by the polymer. This led to a wider molecular weight distribution compared to that of the initial substrates [38]. The yarn with the highest polydispersity was PET-nTiO_2_-A with a value of 3.2.

The rate of polymer degradation can be defined as the rate of scission of the molecular chains and is represented by Equation (1) [39]:

(1) $$\frac{1}{{\bar{M}}_{\mathrm{nt}}}=C+k_{n}t$$

where $C=1/{\bar{M}}_{n0}$, ${\bar{M}}_{n0}$, and y ${\bar{M}}_{\mathrm{nt}}$ are the number of average molecular weights at time t = 0 and t = t, respectively, k_n_ is the rate constant based on the average molecular weight, and t is the thermal degradation time.

From the plots of $1/{\bar{M}}_{\mathrm{nt}}$) vs. exposure time, it was observed that values obtained from correlation coefficients (R^2^) are higher than 0.94 for all cases, which would validate the assumption that the degradation of the polymer is mainly due to a scission of the molecular chains. Table 3 summarizes the regression coefficients of the degradation kinetics plots. The increasing order of the degradation kinetic constant K_n_ according to its magnitude is PET-nTiO_2_-R < PET-µTiO_2_-R < PET-µTiO_2_-A < PET = PET-nCaF_2_ < PET-nTiO_2_-A < PET-nSiC.

**Figure 4:**
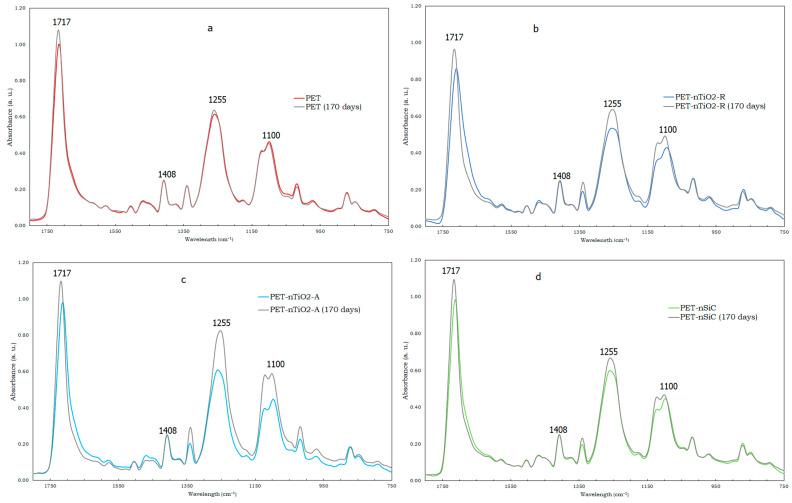
Normalized FTIR–ATR spectra of unexposed and exposed samples after 170 days of accelerated exposure: (**a**) pure PET, (**b**) PET-nTiO2-R, (**c**) PET-nTiO2-A, and (**d**) PET-nSiC. The 1408 cm^−1^ band was used for normalization.

In order to relate the decrease in the molecular weight of the substrates to changes in their chemical composition, FTIR–ATR was carried out. The infrared spectra of pure PET, PET-nTiO_2_-R, PET-nTiO_2_-A, and PET-nSiC are shown in Figure 4. The intensity of the spectra was normalized with a reference peak, the IR band at 1408 cm^−1^, since it is assigned to the benzene ring of PET, which is not expected to be affected by aging [40,41,42] This normalization method allows a proper comparison of the absorption intensity of characteristic bands showing some structural changes during accelerated aging in the 750–1800 cm^−1^ range. An increase in the intensity of characteristic peaks of PET was noted, that is, in the aromatic ester C=O stretch (1717 cm^−1^), aromatic ester C-C-O stretch (1255 cm^−1^), and aromatic ester O-C-C stretch (1100 cm^−1^) [43]. According to a previous study [42], these peaks are due not only to the original PET substrates by themselves but are also the result of the presence of the carbonyl groups in the degradation products. In the case of PET (Figure 4a), the highest increase was observed in the C=O stretch in PET-nTiO_2_-R (Figure 4b), which had the lowest reduction in the molecular weight and a greater raise noted in the three mentioned peaks, and finally, in PET-nTiO_2_-A (Figure 4c) and PET-nSiC (Figure 4d), which were the samples with the highest reduction in the molecular weight, the increase was more evident in the one containing TiO_2_.

**Figure 5:**
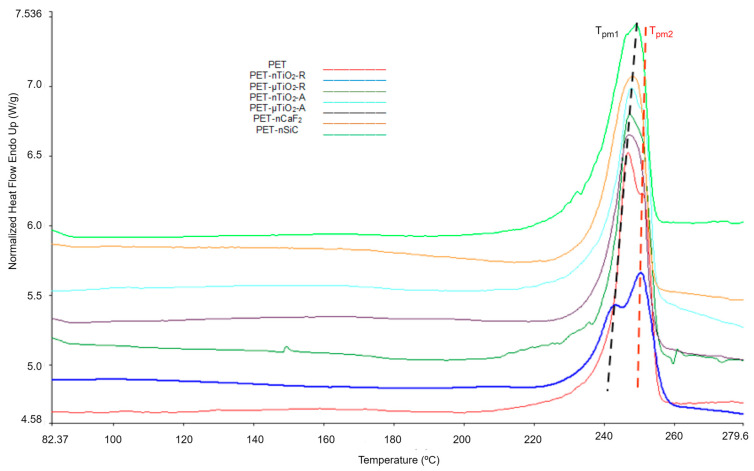
Thermograms corresponding to the first heating of the yarns after 170 days of exposure.

For the study of the variation in the yarn microstructure during the exposure time, DSC melting endotherms of the first heating were analyzed. Figure 5 shows the thermograms corresponding to the study of the yarns after 170 days of exposure. It was observed that the onset of the melting occurred at different temperatures and that, in all cases, there were two crystal distributions. The “shoulder” T_pm1_ corresponds to lower melting temperatures with smaller crystal sizes and T_pm2_ corresponds to high melting temperatures and larger crystal sizes. For all temperatures, the standard deviation (SD) corresponds to the average of the 10 values obtained from each of the extractions, from 0 to 170 days.

**Figure 6:**
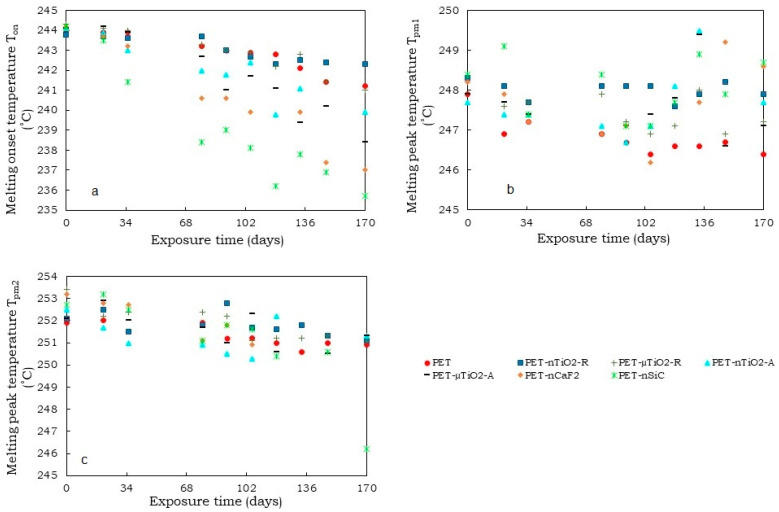
Variation in temperatures as a function of the exposure time (1st heating): (**a**) melting onset temperature (T_on_), (**b**) melting peak temperature (T_pm1_), and (**c**) melting peak temperature (T_pm2_).

As for the melting onset temperature (Figure 6a), a decrease was observed during the exposure time for all cases. This could be due to the breakage of the molecular chains causing a diminution in the size of the crystals, which melted at a lower temperature [44]. For PET-nCaF_2_ and PET-nSiC, the decrease in the melting onset temperature was more noticeable compared to the yarns containing TiO_2_. This was seen at a greater level in PET-nSiC yarns.

Regarding the temperature of the shoulder T_pm1_ (Figure 6b) and main melting peak T_pm2_ (Figure 6c), for pure PET they remained constant since the standard deviation (SD) of the average values did not exceed ± 0.8 °C. This behavior was also observed in the four yarns with TiO_2_. For the case of PET-nCaF_2_ and PET-nSiC, it could be seen that the temperatures of the main melting peak T_pm2_ remained constant throughout the exposure time since the standard deviation of the average values was not greater than ± 0.9 °C. Nevertheless, a decrease in the shoulder temperature T_pm1_ next to the main melting peak was observed in both cases. For PET-nCaF_2_, there was an overlapping of the shoulder T_pm1_ and main melting peak temperature T_pm2_ from 133 days of exposure. 

**Figure 7:**
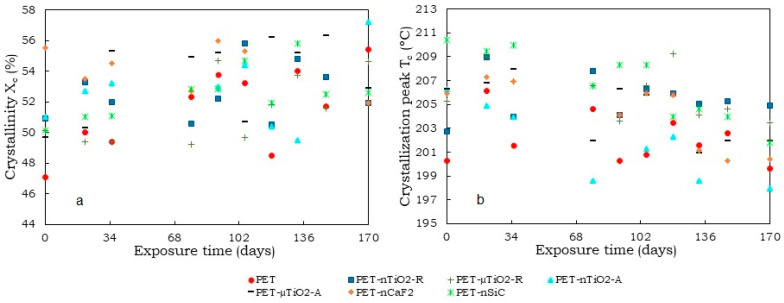
(**a**) Crystallinity of yarns (1st heating) and (**b**) Crystallization peak of yarns as a function of the exposure time.

In terms of crystallinity (Figure 7a), an increasing trend was observed in the case of pure PET, which could be due to a decrease in the amorphous part of the material. The temperature at which the aging of the samples was carried out (50 °C) was lower than the glass transition of PET (70–80 °C) [44,45] and as the water in the medium was not a plasticizer of the polyester [46], no intense heat treatment that enhanced the crystallinity of the substrates would have taken place at that temperature. An increase in crystallinity was also noted for the TiO_2_ yarns, which could be due to the decrease in the amorphous part of the material for the reasons discussed above. The crystallinity of PET-nCaF_2_ at the end of the exposure time was higher than that of PET-nSiC, and no significant increase in crystallinity was observed compared to the unexposed substrates.

The average percent of crystallinity after 170 days of exposure for the yarns was 51.5 ± 2.7% for PET, 52.5 ± 1.8% for PET-nTiO_2_-R, 51.4 ± 2.2% for PET-µTiO_2_-R, 52.7 ± 2.7% PET-nTiO_2_-A, and 52.7 ± 2.6% for PET-µTiO_2_-A, which, in this case, indicates that the particle size and crystalline form of the particles have not greatly influenced the crystallinity of the substrate at aging conditions, 54.0 ± 1.5% PET-nCaF_2_ and 52.5 ± 1.7% PET-nSiC.

Regarding the nonisothermal crystallization peak (T_c_) (Figure 7b), no significant variations were observed in pure PET because of the exposure in the climatic chamber, and the differences were attributable to random experimental errors. For the blends with particles, an increment of T_c_ in the unexposed yarns with nanoparticles was seen, which meant that they acted as nucleating agents [44,47] In previous studies [14,15,19], it was found that the use of Licowax-E (montane acid ester with multifunctional alcohols) as a dispersant influenced such behavior of the ceramic particles. In the case of exposed yarns, a similar response was found in all of them. A tendency for the temperature of crystallization (T_c_) to decrease at higher exposure times was observed. This may be a consequence of a diminution in the concentration of the nucleating agents in the molten polymer, resulting in a lower crystallization temperature upon cooling compared to the untreated substrates. This reduction in concentration could be due to the scission of the chains during the aging process, which would make it possible for the ceramic particles to rise to the surface of the yarns, or for a possible migration of the particles to the surface in analogous mode to disperse dyes [48]. In both cases, the particles would be more easily removed with the airflow circulating inside the climatic chamber or removed in the washing before the DSC test.

**Figure 8:**
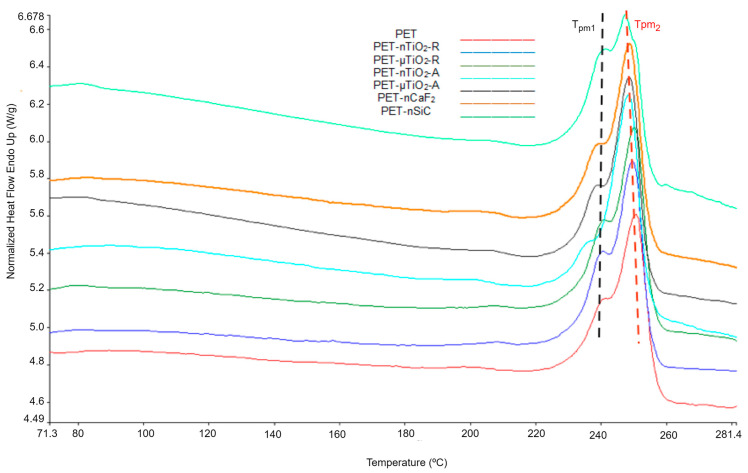
Thermograms of the second heating of the polymer blends after 170 days of exposure.

The melting endotherms of the second heating were analyzed to determine the variation in the properties of polymer blends during the exposure time. The thermograms after 170 days of exposure are shown in Figure 8. Two melting peaks T_pm1_ and T_pm2_ are observed.

**Figure 9:**
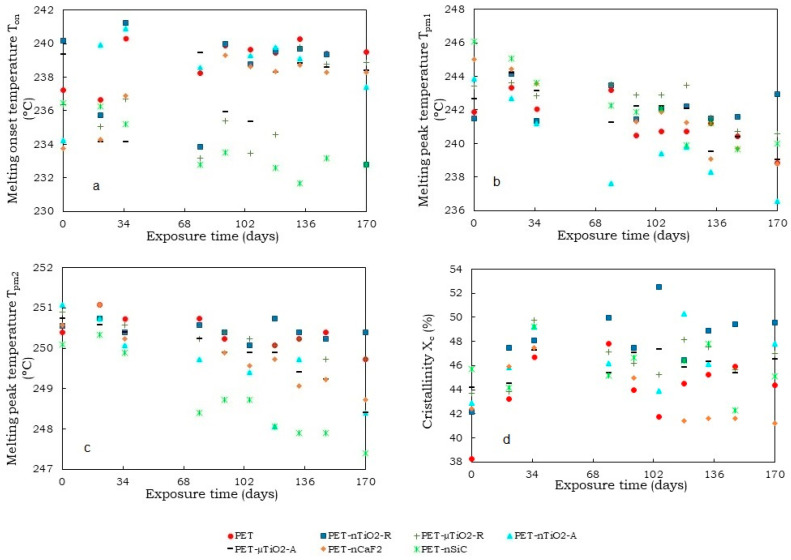
(**a**) Variation in temperatures as a function of the exposure time (2nd heating): (**a**) melting onset temperature (*T_on_*), (**b**) melting peak temperature (*T_pm1_*), (c) melting peak temperature (*T_pm2_*), and (**d**) crystallinity *(X_c_*).

As for the melting onset temperature T_on_ (Figure 9a), a slight increase was observed with respect to the value for pure PET. Nevertheless, the standard deviation was ± 0.4 °C, which was within the experimental error of the test. For TiO_2_-containing polymer blends, no clear trend was observed during the exposure time; however, there was a tendency for this value to increase. For the PET-nCaF_2_ and PET-nSiC blends, the changes were more evident. In the first case, an increase in onset temperature was noted of about 4 °C, and in the second case, a reduction of about 4 °C was seen. This would indicate that, for these two cases, the presence of the nanoparticles has influenced the size of the polymer crystals.

A slight decrease in the shoulder temperature (T_pm1_) (Figure 9b) was seen with respect to the original PET during the course of exposure time, though it was within the experimental error of the test. The temperature of the main peak (T_pm2_) (Figure 9c) remained practically constant (SD ± 0.4 °C). In addition, an increase in crystallinity (Figure 9d) of PET was observed, possibly due to the decrease in the amorphous part of the material [47] because of the influence of UV radiation, temperature, and humidity. 

For TiO_2_ blends, the temperature of the main peak (T_pm2_) was similar during the exposure and was around 250 °C. For all four cases, an increase in crystallinity was observed with respect to the unexposed substrates as a result of the nucleating effect of the ceramic particles used. After 170 days of exposure, the most crystalline was PET-nTiO_2_-R with an average value of 48.2 ± 2.7%, followed by PET-nTiO_2_-A with 46.7 ± 2.4%, PET-µTiO_2_-R, 46.4 ± 1.8%, and PET-µTiO_2_-A with 46.0 ± 1.1%, which indicates that, for this case, the nanoparticles, due to their size, have allowed the formation of a greater quantity of crystals as there are more crystallization nuclei available [49]. 

Concerning the blends PET-nCaF_2_ and PET-nSiC, the results showed that the main peak (T_pm2_) remained constant throughout the treatment time (SD = 0.7). As for crystallinity, in both cases, the values at the end of the treatment remain similar to those of the unexposed blends with 43.7 ± 2.6% and 45.8 ± 2.0%, respectively.

Comparing the DSC melting endotherms of the first and second heating, some differences were noted. Regarding the presence of two melting peaks, which are observed in Figure 5 and Figure 8 (or a main peak and a shoulder), it was observed that in the first heating, the proportion of smaller crystals (lower melting temperature) was higher than the larger ones (higher melting temperature), while in the second heating, the proportion of larger crystals was higher than the smaller ones. These differences could be due to the cooling rate. In the case of spinning, the yarn collection speed on the winding machine was 3000 m/min, which implies fast cooling and, therefore, the formation of a greater quantity of smaller crystals. On the contrary, in the calorimetric test, the cooling speed was 20 °C/min, considerably lower than in the first case, which enabled the formation of a larger quantity of crystals of larger size.

Regarding the onset melting temperatures (T_on_) obtained in the first second heating (Figure 6a and Figure 9a), a decrease in the onset temperature in the second heating was noted, which could be a consequence of the existence of smaller crystals that could have formed in the nonisothermal crystallization, which began to melt at lower temperatures.

The percentages of crystallinity of the mixtures in the first and second heating are shown in Figure 7a and Figure 9d. It was observed that the crystallinity in the second heating was lower, which could be because the substrate, once melted, has neither been cooled with stretching (not oriented) nor subjected to other conditions that allow the increase in its crystallinity, unlike the first heating, in which a previous stretching of the yarns had been performed at 190 °C at a 1:2 ratio.

The average values obtained from the tensile tests of the yarns before their exposure in the climatic chamber are shown in Table 4. It can be observed that PET-nTiO_2_-A was the yarn with the highest count (18.5 ± 0.6 tex) and the lowest count corresponded to PET-µTiO_2_-R (13.3 ± 0.1 tex). Although the yarns with ceramic particles should have a higher count compared to the original PET (16.0 ± 0.2 tex), the conditions of preparation of the blends, their subsequent spinning, and the loss of filaments in the drawing could have influenced the weight of the yarns and, therefore, the final count.

Regarding tenacity, in all cases, the values obtained were lower than those of pure PET (35.86 cN/tex), which could be a consequence of the decrease in molecular weight caused by the inclusion of ceramic particles and their high concentration in the substrates. Previous studies have found that increasing the number of ceramic particles, specifically TiO_2_, to a value higher than 0.33 % in Nylon yarns results in a reduction in tensile strength. High particle concentrations, which, for this case, are in the range of 1.8 wt.% to 2 wt.%, cause interactions and agglomerations, leading to a reduction in the contact surface between the particles and the matrix. In addition, these particles could also act as stress concentration sites and, therefore, when subjected to tensile deformation, the larger particles induce higher stress concentrations in the matrix and induce a reduction in impact energy, which results in the initial breakage of the material appearing earlier [50,51,52,53,54].

**Figure 10:**
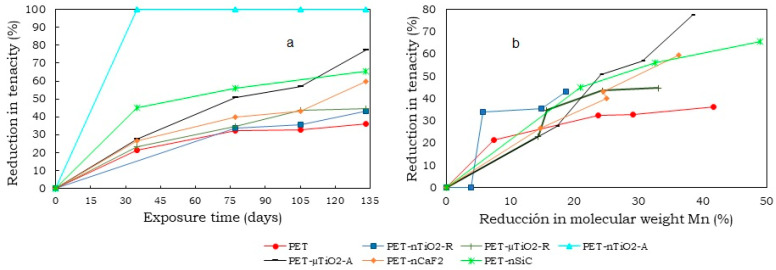
(**a**) Reduction in tenacity after 135 days in the climatic chamber and (**b**) Reduction in tenacity as a function of reduction in molecular weight.

The reductions in tenacity after 135 days of exposure in the climatic chamber are shown in Figure 10. As can be seen, all of the yarns experienced a reduction in their tenacity greater than that of pure PET, which was a consequence of the high number of ceramic particles in the composite materials and the breakage of the molecular chains that constituted the substrate [50]. The yarns in descending order according to their tenacity would be as follows: PET > PET-nTiO_2_-R > PET-µTiO_2_-R > PET-nCaF_2_> PET-nSiC > PET-µTiO_2_-A > PET-nTiO_2_-A.

**Figure 11:**
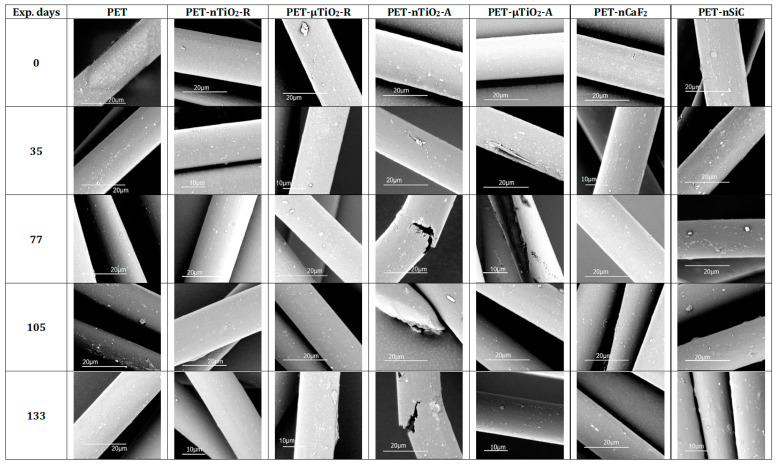
Micrographs of yarns.

In the case of the PET-nTiO_2_-A yarn, it was not possible to perform the dynamometric test due to its high brittleness, which is why a 100% reduction in its resistance was considered after 35 days of exposure in the climatic chamber. In particular, the brittleness of the yarn could be the result of exposure to the climatic chamber and/or the high content of particles. On one hand, for an estimated 13% reduction in molecular weight, the yarns with microparticles showed a 20% reduction in strength, less than the reduction in pure PET, which was approximately 25%. On the other hand, PET-nTiO_2_-R lost 35% and PET-nTiO_2_-A lost 100% of its tensile strength. It could be observed that, over time, PET-µTiO_2_-A significantly decreased in strength compared to PET-µTiO_2_-R because of the brittleness, as observed in Figure 11.

When PET-nTiO_2_-A yarn had lost 100% of its tensile strength with a molecular weight reduction of 13%, PET-nTiO_2_-R lost 35% of its strength, PET-nSiC and pure PET lost 25 %, and PET-nCaF_2_ had lost 20%. Subsequently, the loss of the PET-nSiC yarn strength increased significantly. For a 30% reduction in molecular weight, a 33% reduction in PET tensile strength was observed, 50% for PET-nCaF_2_, 53% for PET-nSiC, and 100% for PET-nTiO_2_-A. 

The micrographs corresponding to the samples observed after 0, 35, 77, 105, and 133 days of exposure are presented in Figure 11. In the case of pure PET yarns, oligomers could be seen on the surface. As for the yarns with ceramic particles, some agglomerations were observed on the surface, which, at first sight, could not be differentiated as oligomers or particles. Nevertheless, the use of the dispersant, the shear forces applied for blending, and the melt spinning technique made it possible to obtain a composite with a good dispersive and distributive blending so that it would not be possible that the agglomerates were particles and would rather be oligomers. Moreover, as mentioned before, this effect was also observed in pure PET that did not contain any charge, which indicated that they were not particles.

In the yarns with nano and microparticles of anatase TiO_2_, fractures caused by brittleness due to their exposure in the climatic chamber were observed, which could lead to the loss of their mechanical properties.

## 4. Conclusions

The presence of ceramic nano and microparticles in the degradation of PET yarns subjected to accelerated aging in a climatic chamber (1.4 W/m^2^ UVA, 50 °C 50% RH) for 170 days was assessed. The following aspects can be highlighted:

1.  A decrease in the molecular weight of all yarns that have been subjected to exposure in the climatic chamber was noted. This could be due to the excision of the molecular chains that formed the polymeric matrix. On one hand, the reduction was more evident in yarns with anatase TiO_2_ and SiC nanoparticles (about 50%), possibly due to their higher specific surface area that could have caused the ultraviolet radiation to produce a larger degradation. On the other hand, the rutile TiO_2_ nanometric had only a reduction of 25.3%.
2.  A trend of increasing crystallinity in the pure PET yarns was observed over time, which could be due to a decrease in the amorphous part of the material. Moreover, the particle size and crystalline form of the ceramic particles did not increase the crystallinity compared to yarns that had not been exposed. Contrarily, in the case of polymer blends containing TiO_2_, unlike CaF_2_ and SiC, there was an increase in crystallinity because of the nucleating effect of the ceramic particles used. In this case, the nanoparticles, due to their size, allowed the formation of a greater number of crystals.
3.  Although an increase in nonisothermal crystallization temperature was observed in yarns with ceramic particles compared to pure PET, a tendency was found for it to decrease at higher exposure times because of a possible decrease in the concentration of the nucleating agents in the polymer melt because of the exposure in the climatic chamber.
4.  Finally, a reduction in tensile strength greater than that of the original PET was observed for all of the yarns that were subjected to the aging treatment. This could be a consequence of the high number of ceramic particles present in the composite materials and the breakage of the molecular chains of the substrate. The yarn with the anatase TiO_2_ micrometric showed the highest brittleness, which also experienced the greatest decrease in its molecular weight.

This study provides insight into the evolution of the properties of PET-based nano- and microcomposites and might be helpful when selecting materials for specific applications that require continuous exposure to UV radiation, humidity, and temperature, which is of great interest from an industrial point of view.

## Figures and Tables

**Table 1:** Multifilament yarns used in this study.

Substrate	Ceramic Particle	Ceramic Particle (%)	Dispersant (%)	Name of the Sample
PET	-	0	0	PET
	TiO_2_ rutile nm	1.8	1.8	PET-nTiO_2_-R
	TiO_2_ rutile µm	2.0	2.0	PET-µTiO_2_-R
	TiO_2_ anatase nm	2.0	2.0	PET-nTiO_2_-A
	TiO_2_ anatase µm	2.0	2.0	PET-µTiO2-A
	CaF_2_ nm	2.0	2.0	PET-nCaF_2_
	SiC nm	2.0	2.0	PET-nSiC

**Table 2:** TiO_2_ particle characteristics provided by the manufacturer.

Particle type	Rutile Nanometric		Rutile Micrometric		Anatase Nanometric	Anatase Micrometric	
Commercial reference	TiO_2_ MT-500HD Tayca Corporation		TiO_2_ KRONOS 2360		TiO_2_ AMT-600 Tayca Corporation	TiO_2_ KRONOS 1071	
Particle size (nm)	30		190		30	220	
Specific surface area (m^2^/g)	48		13–17		52	9–11	
Composition (%)	TiO_2_	85	TiO_2_	≥ 92	TiO_2_ 80–98	TiO_2_	≥96
	Al_2_O_3_	1–15	Al_2_O_3_	3–3.8		Al_2_O_3_	1–1.2
	ZrO_2_	1–10	SiO_2_	2.4–3		SiO_2_	0.5–0.7
			C	0.18–0.2		P_2_O_5_	0.3–0.4
						C	0.15–0.25
Melting temperature (°C)	1843						

**Table 3:** Coefficients of yarn degradation kinetics.

Sample	K_n_ (mol/kg·day) (10^−4^)	C (mol/kg) (10^−2^)	R^2^
PET	2.0 ± 0.2	3.4 ± 0.2	0.946
PET-nTiO_2_-R	0.9 ± 0.1	4.6 ± 0.1	0.941
PET-µTiO_2_-R	1.7 ± 0.1	4.2 ± 0.1	0.949
PET-nTiO_2_-A	2.6 ± 0.1	4.0 ± 0.1	0.984
PET-µTiO_2_-A	1.9 ± 0.1	4.3 ± 0.1	0.982
PET-nCaF_2_	1.9 ± 0.1	4.9 ± 0.1	0.970
PET-nSiC	2.9 ± 0.1	4.9 ± 0.3	0.947

**Table 4:** Yarns count, tenacity, and elongation before exposure to the climatic chamber.

Sample	Count (tex)	Tenacity		Elongation	
		Value (cN/tex)	CV (%)	Value (%)	CV (%)
PET	16.0 ± 0.2	35.9	4.4	23.1	9.6
PET-nTiO_2_-R	17.2 ± 0.2	17.9	12.2	24.5	20.6
PET-µTiO_2_-R	13.3 ± 0.1	33.1	5.5	20.4	12.7
PET-nTiO_2_-A	18.5 ± 0.6	21.4	8.0	27.1	14.4
PET-µTiO_2_-A	16.9 ± 0.4	31.0	4.5	21.5	9.7
PET-nCaF_2_	15.3 ± 0.1	28.5	7.5	21.9	13.8
PET-nSiC	16.4 ± 0.9	24.3	4.7	23.0	7.3

## Data Availability

The data presented in this study are available on request from the corresponding author. The data are not publicly available due to privacy.
